# COVID-19 Vaccine Hesitancy and Misinformation Endorsement among a Sample of Native Spanish-Speakers in the US: A Cross-Sectional Study

**DOI:** 10.3390/healthcare12151545

**Published:** 2024-08-05

**Authors:** Elizabeth A. Carosella, Maxwell Su, Marcia A. Testa, Guglielmo Arzilli, Alice Conni, Elena Savoia

**Affiliations:** 1 Emergency Preparedness Research Evaluation & Practice Program (EPREP), Division of Policy Translation & Leadership Development, Harvard T.H. Chan School of Public Health, 677 Huntington Avenue, Boston, MA 02115, USA; ecarosella@mathematica-mpr.com (E.A.C.); masu@hsph.harvard.edu (M.S.); testa@hsph.harvard.edu (M.A.T.); 2 Mathematica Policy Research, Mathematica, Princeton, NJ 08540, USA; 3 Department of Biostatistics, Harvard T.H. Chan School of Public Health, 677 Huntington Avenue, Boston, MA 02115, USA; 4 Department of Translational Research and New Technologies in Medicine and Surgery, University of Pisa, 56126 Pisa, Italy; guglielmo.arzilli@phd.unipi.it; 5 Department of Medical and Surgical Sciences, University of Bologna, 40126 Bologna, Italy; alice.conni@studio.unibo.it

**Keywords:** COVID-19, vaccination, misinformation, Spanish speaking

## Abstract

Research on COVID-19 vaccine hesitancy and misinformation endorsement among Spanish-speaking Americans is limited. This cross-sectional study used a Spanish-language survey from May–August 2021 among 483 Spanish speakers living in the US and Puerto Rico. We applied multivariable Poisson regression with robust error variances to assess the association between independent variables and binary outcomes for vaccine acceptance versus hesitance, as well as misinformation endorsement. Vaccine acceptance was associated with COVID-19 risk perception score (PR = 1.7 high vs. low perceived risk), opinion of government transparency (PR = 2.2 very transparent vs. not transparent), and trust in vaccine information (PR = 1.8 high vs. low). There was also an interaction between time spent on social media and social media as a main source of COVID-19 information (*p* = 0.0484). Misinformation endorsement was associated with opinion about government transparency (PR = 0.5 moderately vs. not transparent), trust in vaccine information (PR = 0.5 high vs. low trust), social media impact on vaccine confidence (PR = 2.1 decreased vs. increased confidence), distrust vaccines (PR = 1.9 distrust vs. trust), using vaccine information from Facebook (PR = 1.4 yes vs. no), and time spent on social media by those using social media as main source of COVID-19 vaccine information (*p* = 0.0120). Vaccine acceptance in respondents with high misinformation endorsement scores was 0.7 times those with low scores. These findings highlight the importance of effective information dissemination, the positive role of social media, and government transparency in boosting vaccine uptake among Spanish speakers in the US.

## 1. Introduction

Immunization is the primary public health strategy to prevent severe illness and death from COVID-19. However, vaccine uptake has been inconsistent across the United States, with widely documented racial and ethnic disparities [1]. The Hispanic population in the United States has been disproportionately impacted by the COVID-19 pandemic, with a 1.5 times greater risk of being infected and a 1.7 times greater risk of dying from COVID-19 compared to the white population [2]. Despite this, during the pandemic, Hispanic adults waited longer on average to get vaccinated than white adults [3]. Hispanic workers also comprise a larger proportion of essential workers than white workers, putting them at higher risk of getting infected. In addition, this population is more likely to suffer from preexisting health conditions such as obesity, diabetes, and heart disease, increasing the likelihood of complications from SARS-CoV-2 infection [3,4]. Despite persistent disparities in COVID-19 infection and vaccine uptake among Hispanic adults, research to understand vaccine hesitancy among Hispanic, native Spanish-speaking Americans has been limited.

It is worth noting that, although at the start of the vaccination campaign, Hispanic adults were less likely than white adults to be immunized, vaccination rates among Hispanic individuals rose during 2022 [1]. In November 2022, the Centers for Disease Control and Prevention (CDC) estimated that 87.5% of Hispanic adults had received at least one dose of the vaccine, as inferred from the CDC COVID Data Tracker published in May 2023 [5]. However, despite this high proportion receiving the primary vaccination dose, there has been low uptake of the booster dose, which protects against severe illness and death from the Omicron variant [6]. Only 12.7% of Hispanic individuals have received an updated bivalent booster dose, less than half of the percentage of white adults [5].

The Hispanic population makes up a substantial and growing portion of the U.S. population. The U.S. Census Bureau reported that Hispanic Americans are the second largest racial or ethnic group in the U.S. Nearly one in five Americans is Hispanic, over 62 million people in the country [7]. However, a systematic review of vaccine hesitancy found that most surveys that reported race and ethnicity had most respondents from the white, non-Hispanic population [8]. Furthermore, many studies using qualitative approaches lumped together Hispanic individuals with African Americans, being unable to identify differences between these two very diverse groups. Other recent studies have not found associations among Hispanic populations for COVID-19 vaccine access and hesitancy issues [9,10,11,12]. As such, research focusing on this population sub-group is limited.

Among factors potentially associated with vaccine hesitancy in this population, language barriers should be considered. As reported by the Washington Post, forty percent of Hispanic Americans use Spanish for their primary communication, and only 30% consider themselves bilingual [13]. Nearly 42 million people, or 13.5% of the US population, are native Spanish speakers [14], making this group larger than the entire population of Canada [15]. Nevertheless, much of the health information produced by federal, state, and local governments is published and communicated in English, and past research has found that barriers to vaccination among Hispanic respondents include poor Spanish translations of vaccine materials [4].

Regarding other potential factors associated with vaccine hesitancy, research on the relationship between demographic factors and vaccine hesitancy in the U.S. has identified sex, race, age, education, and income status as determining factors of both low and high COVID-19 vaccine uptake in different population samples [8]. In addition, numerous studies document the role of information channels and exposure to misinformation on vaccine hesitancy [16,17,18,19,20].

Social media is a well-documented vehicle for the spread of misinformation, though the volume of misinformation may vary by platform. U.S. adults who obtained information from social media were more vaccine-hesitant than those who had not used those platforms [21]. A total of 86% of Hispanic adults report using YouTube, and 66% use Facebook, the highest proportion of any racial or ethnic group in the country [22]. Hispanic adults are more inclined to use social media for COVID-19 information compared to white adults [13]. Yet, social media companies perform content moderation primarily in English and have acknowledged their inability to identify and remove misinformation in Spanish accurately [4].

Beliefs in conspiracy theories and misinformation about COVID-19 vaccines are also associated with lower vaccine acceptance [23,24]. Conversely, trust in information has been found to increase vaccine acceptance [21], and trust in channels of information has been shown to impact how an individual may act upon the information received [25].

Among other factors, social channel use preference and trust in institutions are strongly correlated and independently predict vaccine hesitancy [26]. Trust in the government was reported to be a predictor of vaccine hesitancy during the COVID-19 vaccination campaign [27]. Therefore, trust in vaccine information within the Hispanic population, considering both the institutional source of information and the channel through which the information is received, are important factors worth exploring to better understand vaccine hesitancy in this population sub-group. Previous research shows the importance of tailoring vaccine campaigns and interventions to socio-economic and cultural contexts, and as such, it is important to identify factors associated with the specific informational needs and characteristics of specific population sub-groups [28].

To fulfill these knowledge gaps, we conducted research focusing on the Spanish-speaking population in the US, focusing on factors associated with vaccine hesitancy and misinformation endorsement related to the COVID-19 vaccine within this group. This study aimed to explore (1) socio-demographic and behavioral determinants of COVID-19 vaccine acceptance, (2) socio-demographic and behavioral determinants of COVID-19 vaccine misinformation endorsement, and (3) to test the hypothesis that misinformation endorsement is negatively associated with vaccine acceptance among Spanish speakers.

## 2. Materials and Methods

### 2.1. Study Design

This cross-sectional study was implemented by surveying a purposive sample of 500 adult Spanish speakers across the United States between 21 May and 28 August 2021. The sample was formed using crowdsourcing technology via the online survey platform Pollfish to include all panel respondents having this language characteristic [29], which pays mobile application developers to display and promote the surveys to their users. Respondents indicated Spanish as their primary language, and the survey was implemented in Spanish. A screening question was used to identify respondents who either had not received any COVID-19 vaccine or had only received the first dose of a two-dose vaccine regimen. Anyone who was fully vaccinated was deemed ineligible to participate in the survey. The Spanish version was translated from English and backtranslated into English for validity purposes. Before implementation, the survey underwent cognitive testing with nine individuals. For a copy of the entire questionnaire in both languages, see File S1. The Harvard Chan School Institutional Review Board (IRB) deemed the study protocol and survey instrument exempted on 8 December 2020 (protocol #20-2032). Participants were asked to consent to participate in the study immediately before starting the survey. As the minimum amount of time to thoughtfully complete the survey was tested to be three minutes, we used this time criteria as a method for data quality assurance. We removed questionnaires that were completed in less than three minutes. A total of 17 questionnaires were removed, leaving a final sample size of 483 respondents.

### 2.2. Dependent Variables

The study analyzed two dependent variables: vaccine acceptance and misinformation endorsement.

Vaccine acceptance: Participants were asked about their willingness to receive a COVID-19 vaccine if it were made available to them for free within two months. The response choices included “very likely”, “somewhat likely”, “would not get it now, but would consider it in the future”, “not sure”, “somewhat unlikely”, and “very unlikely”. The six response options were categorized into a binary dependent variable named “vaccine acceptance”, with “very likely” and “somewhat likely” responses categorized as acceptant, and all other responses categorized as hesitant.

Misinformation endorsement was measured by asking respondents to rate the extent to which they disagreed with the following statements; the topics were chosen by reviewing Newsguard monthly reports released during the pandemic [30]:

1. You cannot contract COVID-19 from the vaccine.
2. There are no toxic ingredients in the vaccine that are harmful to your health.
3. The vaccine cannot alter your DNA.
4. The vaccine cannot cause infertility.
5. The vaccine cannot cause other illnesses.
6. The rapid production of the vaccine did not compromise its security.
7. Governments are not going to use the vaccine as a tool to limit our civil rights (right of assembly, right of movement, right of religion, etc.).

Responses to each question were recoded as “totally agree” = 1, “agree” = 2, “somewhat agree” = 3, “I am not sure” = 4, “somewhat disagree” = 5, “disagree” = 6, and “strongly disagree” = 7. From the responses to these seven items, we generated a composite variable describing the construct of misinformation endorsement based on the result of a factor analysis. The Kaiser–Meyer–Olkin (KMO) measure of sampling adequacy and Bartlett’s test of sphericity were used to test the suitability of the data for factor analysis [31]. A factor analysis with the principal factor extraction method found a single factor that had an eigenvalue greater than one supporting the assumption that the seven items measure a unidimensional construct. A KMO value of 0.88 and Bartlett’s *p* < 0.0001 showed the suitability of performing a factor analysis on these items. Cronbach alpha was 0.83, demonstrating the items had moderately high internal consistency. A misinformation endorsement scale score was generated as the sum of the seven items, and the binary-dependent variable of misinformation endorsement was created as the top quartile of scores versus the lower three quartiles. In addition, a four-category misinformation endorsement variable was also created from the quartiles of the misinformation endorsement scale score and used as an independent variable in the vaccine acceptance models.

### 2.3. Independent Variables

#### 2.3.1. Socio-Demographics

We explored socio-demographic variables previously identified as related to hesitancy towards vaccines and health-related behaviors. These were age, gender, and level of education. Food insecurity was measured by asking respondents whether they had worried about not having enough money to buy food in the last year (yes/no), as well as how frequently they had worried about running out of money for food within the last 3 months (often/sometimes/never). Regions of U.S. residency were the Midwest, Northeast, South, and West. We also evaluated the self-reported comorbidities.

#### 2.3.2. Main COVID-19 Information Source

Respondents were asked to choose up to three communication channels from which they had received most of their information about the COVID-19 vaccine. Seven binary variables were created to indicate whether their main source of COVID-19 vaccine information came from social media (Facebook, YouTube, Twitter, Instagram, TikTok, other), TV, radio, newspaper, any traditional media, traditional media in English or traditional media in non-English languages. Since respondents were allowed to choose up to three sources, these indicator variables were not mutually exclusive.

#### 2.3.3. Information from Individual Social Media Platforms and Multiplicity of Platforms

Indicator variables for whether respondents received information about the vaccine using social media and what specific platform they used were created. A question asked respondents whether they received any information about the COVID-19 vaccine from the social media channels of Facebook, Twitter, YouTube, Instagram, or TikTok. Six binary variables indicating whether the respondent received information about the COVID-19 vaccine from such channels were generated. A variable named “number of social media channels” was created from a count of the number of social media platforms from which the respondent received information about the COVID-19 vaccine.

#### 2.3.4. Time Spent Online

To assess a potential dose–response relationship between social media exposure and vaccine acceptance, we asked respondents how much time they spent on social media on average. Response options were every day three for hours or more; every day between 1 and 3 h; every other day; not often; and never. For modelling the interaction between time spent online and social media as an information source, the categories for every other day, not often, and never were combined when the cross-tabulation between the variables in the interaction and the outcome led to small or empty cell counts.

#### 2.3.5. Trust in COVD-19 Vaccine Information

This was assessed by examining the answers to the question “How much do you trust the information you received so far about the COVID-19 vaccine?” with response options: not at all, very little, somewhat, and a lot. Not at all, and very little were grouped into a category labelled “low trust”, creating an ordered categorical variable of “trust” with three levels: low trust, some trust, and high trust. We compared this with an indicator variable for respondents who reported that they trusted social media to obtain information about COVID-19.

#### 2.3.6. COVID-19 Risk Perception Score

COVID-19 risk perception was assessed by requesting participants to indicate how concerned they were about acquiring COVID-19 either in their workplace or in settings outside of work, as well as their worry regarding the possibility of transmitting the virus to family members or friends. The responses to these questions were coded as “not concerned” = 1, “somewhat concerned” = 2, and “very concerned” = 3. From these three questions, we generated a composite variable for risk perception based on the results of a factor analysis. The KMO measure of sampling adequacy was 0.70, and Bartlett’s test of sphericity had *p* < 0.0001, which showed the appropriateness of fitting a factor analysis to the three items. The factor analysis using a principal factor extraction method found a single eigenvalue greater than one, resulting in the creation of a unidimensional scale from the sum of the three items with scores ranging from 3 to 9, and higher values indicating a greater level of concern. Cronbach’s alpha for the three items was 0.78, which indicates good internal consistency of the scale. We segmented the overall score along the interquartile range of the distribution to represent low-, middle-, and high-risk perception and used this composite COVID-19 risk perception score as an independent variable in the Poisson regression analyses.

#### 2.3.7. Personal Impact of COVID-19

These three variables included having been positively diagnosed with COVID-19 (yes/no), having a friend or family member who had a severe COVID-19 infection (yes/no), and having a friend or family member who had died of COVID-19 (yes/no).

#### 2.3.8. Prior Vaccination Behaviors

Questions about past behavior were included to understand how broader vaccine attitudes may be associated with COVID-19 vaccine acceptance. Respondents were asked whether they had ever declined a vaccine (other than COVID-19) that was recommended by a health provider (yes/no/no recollection) and, if so, why. Based on a follow-up question that asked for the reason why the respondent declined the vaccine, two additional binary variables were created to indicate whether the respondent had a distrust of vaccines or a prior bad experience with vaccines.

#### 2.3.9. Social Media Impact on Vaccine Confidence

Respondents were asked whether information from social media changed their confidence in the COVID-19 vaccine, with response categories being “increased”, “reduced”, “no change”, and “unsure”. An impact of social media on vaccine confidence predictor was created with these response categories, along with a category for “no information from social media” for subjects who indicated not getting COVID-19 vaccine information from social media on a prior question in the survey.

#### 2.3.10. Opinion about Government Transparency

From a question asking respondents whether they felt the information about the COVID-19 situation from their national government was transparent, the predictor of the opinion of government information was created with response categories of “not transparent”, “moderately transparent”, “very transparent”, and “I don’t know”.

### 2.4. Statistical Analysis

We began by creating descriptive statistics for the sample’s sociodemographic characteristics. We then conducted exploratory analyses to identify associations between each of the two outcomes of interest and potential predictors. To do so, we first fit simple Poisson regression models with robust error variance to estimate prevalence ratios (PR) and 95% confidence intervals (CIs) for the association between the independent variables and the binary outcomes of vaccine acceptance vs. hesitance and misinformation endorsement [32]. We next fit multivariable models for each outcome including independent variables that were significant in the simple models while adjusting for age, gender, and educational attainment. The non-significant (*p* ≥ 0.05) predictor with the largest p-value was removed from the model in a stepwise manner.

To estimate the effect of social media, we also tested for interactions between the amount of time spent on social media, social media as a main information source, social media as a source of vaccine information, and each platform individually in the multivariable models. Finally, the effect of the degree of misinformation endorsement on the prevalence of vaccine acceptance was examined using the four-category misinformation endorsement variable as a predictor in the vaccine acceptance Poisson models. Data were analyzed using the software Stata 17 [33].

## 3. Results

### 3.1. Sample Characteristics

Table 1 presents key sample characteristics with proportions of vaccine acceptance and results of bivariable chi-squared tests of differences in frequencies of acceptant and non-acceptant respondents by key characteristics. The 483 respondents resided in 44 states and the territory of Puerto Rico. Residents of four states made up 55.3% of the total: Texas (17.6%), California (16.4%), Florida (14.3%), and New York (7.0%).

Over a third (36.7%) reported health conditions associated with increased risk of COVID-19 complications, including overweight (13.3%) and obesity (10.1%), hypertension (12.6%), diabetes (14.3%), heart disease (2.3%), being immunocompromised (4.8%), and pulmonary illness (3.3%).

Willingness to receive a COVID-19 vaccine if it were made available to them for free within a two-month period were distributed as follows: 22.8% “very likely”; 15.7% “somewhat likely”; 17.0% “would not get it now, but would consider it in the future”; 15.5% “not sure”; 10.4% “somewhat unlikely”; and 18.6% “very unlikely”. Vaccine acceptant categories “very likely” and “somewhat likely” comprised 38.5%, with all other responses categorized as hesitant (61.5%).

#### 3.1.1. Food Insecurity

Nearly two-thirds (63.4%) had worried about not having enough money for food in the past year. Within the past 3 months, nearly three-quarters of the sample had worried about their ability to buy food, while nearly one-quarter reported worrying “often”.

#### 3.1.2. COVID-19 Experience

Noting that participation in the study was limited to those who were not fully vaccinated, most respondents (70.8%) had not received any vaccine doses. Nearly half expressed no intention of getting vaccinated. As reported in Table 2, sixty-three percent of respondents had a personal experience with COVID-19 infection, with either a family member, friend, or themselves having been previously diagnosed. Over one in five reported a family member or friend had died from COVID-19.

#### 3.1.3. COVID-19 Risk Perception

Perceived risk of COVID-19 infection at work and outside of work was 67.1% and 73.9% for the “somewhat concerned”, respectively. A total of 78.5% reported worry about infecting friends or family. The composite COVID-19 risk perception score had 19.9% of respondents with low-risk perception, 42.4% with middle, and 37.9% with high.

#### 3.1.4. Main COVID-19 Information Source

Traditional media: 88.0% of the sample reported traditional media as a primary source of COVID-19 information. Local TV news channels were the dominant source of COVID-19 information (50.5%) compared with all other sources. Non-English language traditional media were primary information sources for 23.6% of respondents. Of all traditional media users (*n* = 425), 96.0% used English-language sources, 73.2% exclusively used English sources, 26.8% used non-English language sources, and just 4.0% exclusively used non-English sources.

Social media use was high overall. Only 6.4% of respondents never used social media, and 73.3% of respondents used social media every day. Social media was the main source of COVID-19 information for 30.4% of respondents. Of social media platforms, Facebook was the most used (49.9% of the total), followed by YouTube (37.7%), Instagram (35.2%), TikTok (26.5%), and Twitter (20.9%).

#### 3.1.5. Prior Vaccine Behavior

Nearly one-third of the respondents had declined a recommended vaccine at least once in their lives. The most stated reasons for declining, among the 11 possible reasons listed, were lack of information (14.3%)**,** concern about side effects (12.6%), mistrust of vaccines in general (12.2%), not necessary (10.6%), and not effective (10.1%).

#### 3.1.6. Misinformation Endorsement

File S2 describes the proportion of the sample who held various misbeliefs. There was a fair distribution across the three levels of agree, unsure, and disagree; however, a plurality of the sample believed that the vaccine could cause COVID-19 (42.0%) or other diseases (41.2%), contain toxic ingredients (38.7%), and that its safety was compromised by the speed of development (37.5%).

### 3.2. Simple and Multivariable Poisson Regression Analyses

#### 3.2.1. Determinants of Vaccine Acceptance

**Figure 1:**
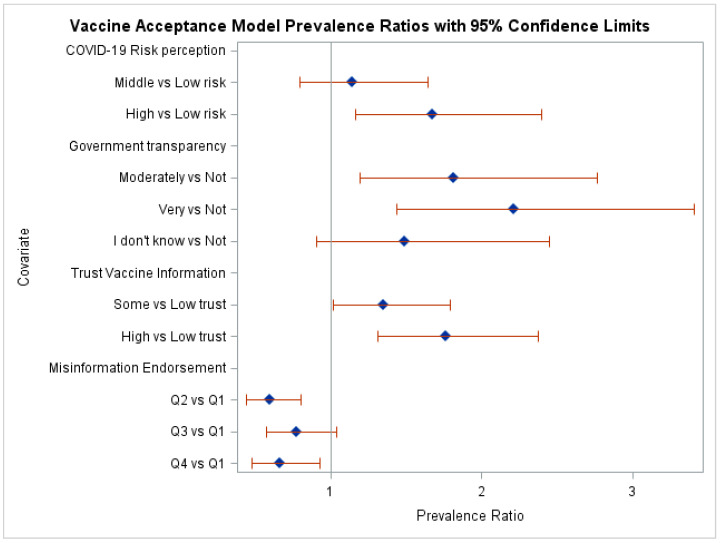
Prevalence ratios and confidence intervals for the covariates in the vaccine acceptance model.

Table 2 presents the results of simple and multivariable Poisson regression models with robust error variance among independent variables and vaccine acceptance. To limit the size of the table, socio-demographic predictors are not included but will be discussed in the text when significant. For categorical predictors with more than two response categories, the table includes the p-value associated with the contrast testing the overall significance of the predictor and only includes prevalence ratios between the reference group and all other groups, rather than all pairwise comparisons. In the simple models, the predictors COVID-19 risk perception, opinion about government transparency, trust in vaccine information, social media impact on vaccine confidence, misinformation endorsement score, indicator for no vaccine information from social media, and trust vaccine information from social media were significant at *p* < 0.05. The multivariable model was initially fitted using predictors that were significant in the simple models along with controls for age, gender, and education level. After removing non-significant predictors in a stepwise manner and testing for significant interactions between the amount of time spent on social media, social media as a main information source, social media as a source of vaccine information, and each platform individually, the final model included COVID-19 risk perception (*p* = 0.0005), opinion about government transparency (*p* = 0.0022), trust in vaccine information (*p* = 0.0008), misinformation endorsement (*p* = 0.0031), and the time spent on social media by social media as a main source of COVID-19 information interaction (*p* = 0.0484). Figure 1 shows the PR for the covariates resulting in the multivariate model.

The prevalence of vaccine acceptance in the high COVID-19 risk perception score group was 1.5 (95% CI: 1.2, 1.8) times the middle-risk group and 1.7 (95% CI: 1.2, 2.4) times the low-risk group. The opinion about the government transparency effect was driven by the “very transparent” and “moderately transparent” groups, having 2.2 (95% CI: 1.4, 3.4) and 1.8 (95% CI: 1.2, 2.8) times the prevalence of vaccine acceptance than the “not transparent” group, respectively. In addition, the proportion of vaccine acceptant in the “very transparent” group was 1.5 (95% CI: 1.0, 2.1) times that of the “I don’t know” group. The trust in vaccine information effect was due to significant differences between all levels of trust, with the “high trust” group having a prevalence of vaccine acceptance 1.8 (95% CI: 1.3, 2.4) times the “low trust” group and 1.3 (95% CI: 1.0, 1.7) times the “some trust” group, while the “some trust” group was 1.3 (95% CI: 1.0, 1.8) times the “low trust” group. Respondents with high misinformation endorsement (fourth quartile of scores) and those in the second quartile were 0.7 (95% CI: 0.5, 0.9) and 0.6 (95% CI: 0.4, 0.8) times more likely to be vaccine acceptant as those with low misinformation endorsement (first quartile), respectively. The significant interaction between time spent on social media and social media as a main source of COVID-19 vaccine information is due to the prevalence ratio of vaccine acceptance between people who use social media as a main source of vaccine information versus those who do not vary across the amount of time spent on social media. This prevalence ratio was 0.6 (95% CI: 0.2, 1.3), 0.8 (95% CI: 0.6, 1.3), and 1.4 (95% CI: 1.0, 1.9) for people who were online less than daily, spent 1–3 h a day online, and spent more than three hours a day online, respectively. This meant the prevalence ratio of vaccine acceptance of those who spent more than three hours a day online was 2.5 (*p* = 0.0469) times that of those who were online less than daily.

#### 3.2.2. Determinants of Misinformation Endorsement

**Figure 2:**
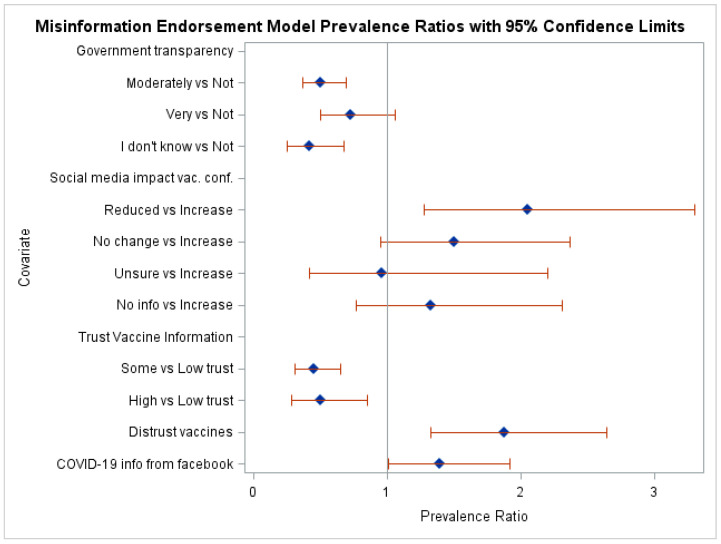
Prevalence ratios and confidence intervals for the covariates in the misinformation endorsement model.

Table 2 also presents prevalence ratios for determinants of misinformation endorsement. Significant predictors in the simple models were having ever declined a recommended vaccine, opinion about government transparency, number of social media channels, trust in vaccine information, social media impact on vaccine confidence, having ever being diagnosed with COVID-19, prior bad experience with vaccines, distrust vaccines, social media as a main COVID-19 information source, and vaccine information from Facebook. Gender and food insecurity during the past three months were also significant predictors in the simple model analysis but are not shown in Table 2. The multivariable model, after removing non-significant predictors in a stepwise manner, retained the following significant predictors: opinion about government transparency (*p* < 0.0001), trust in vaccine information (*p* < 0.0001), social media impact on vaccine confidence (*p* = 0.0148), distrust vaccines (*p* = 0.0003), vaccine information from Facebook (*p* = 0.0428), and the interaction between amount of time spent on social media and social media as a main source of COVID-19 information (*p* = 0.0120), while adjusting for age, gender, and education level. Figure 2 shows the PR for the covariates resulting in the multivariate model.

Regarding the effect of the opinion of government transparency, the prevalence of misinformation endorsement in the “moderately transparent” and “I don’t know” groups were 0.5 (95% CI: 0.4, 0.7) and 0.4 (95% CI: 0.3, 0.7) times the “not transparent” group, respectively. In addition, the prevalence of misinformation endorsement in the “very transparent” group was 1.8 (95% CI: 1.0, 3.1) times the “I don’t know” group.

Differences between levels of trust in vaccine information found that people with “some trust” and people with “high trust” had 0.4 (95% CI: 0.3, 0.7) and 0.5 (95% CI: 0.3, 0.9) times the prevalence of misinformation endorsement, respectively, compared with those with “low trust”.

The impact of social media on vaccine confidence was significant because people in whom social media decreased their vaccine confidence were 2.1 (95% CI: 1.3, 3.3) and 2.1 (95% CI: 1.0, 4.5) times more likely to endorse misinformation than people who reported increased vaccine confidence due to social media and those that were unsure, respectively. People who had a distrust of vaccines had 1.9 (95% CI: 1.3, 2.6) times the prevalence of misinformation endorsement than those who trusted vaccines. People who received t vaccine information from Facebook had 1.4 (95% CI: 1.0, 1.9) times the prevalence of misinformation endorsement than those who did not. For the significant interaction between time spent on social media and social media as a main source of COVID-19 vaccine information, the results show prevalence ratios of misinformation endorsement between those who use social media as a main source of vaccine information versus those who do not were 1.4 (95% CI: 0.6, 3.3), 1.0 (95% CI: 0.6, 1.7), and 0.4 (95% CI: 0.2, 0.7) for people who were online less than daily, 1–3 h a day, and more than three hours a day, respectively. The ratios between prevalence ratios (PRs) for those who spent more than three hours a day online and those online 1–3 h a day compared with those online less than daily were 0.3 (95% CI: 0.2, 0.8) and 0.3 (95% CI: 0.1, 0.8), respectively.

## 4. Discussion

Although it is known that different ethnic groups have different attitudes towards vaccine uptake [34], not many studies have been conducted on COVID-19 vaccine hesitancy focusing exclusively on the Spanish-speaking population in the US [35,36,37,38,39,40,41]. Our study aimed to describe the determinants of vaccine hesitancy and misinformation among Spanish-speaking people who either did not receive both doses of the COVID-19 vaccine or were not vaccinated at all. Our sample included respondents from over 40 US states.

We found that the greater people’s trust in information about the COVID-19 vaccine, the greater their vaccine acceptance. For those using social media as a primary COVID-19 information source, the more time they spend on social media, the higher their vaccine acceptance. Additionally, greater social media use was associated with a decreased likelihood of endorsing misinformation about the vaccine. This result is consistent with research showing that exposure to inaccurate information is concentrated among narrow group of people that are actively searching for such information online [42].

Finally, we found that people who believed in the government being transparent in its communication about the vaccine were more vaccine acceptant and were less likely to endorse vaccine misinformation. Our findings contribute to the ongoing dialogue on effective communication strategies, demonstrating that diverse aspects of communication can significantly affect the success of vaccination policies. Such strategies include the importance of building trust in the public from a message and messenger perspective [43], as well as the impact that social media can play in reinforcing such message [44]. The study by McKinley et al. shows that preference for information through social media and trust are strongly correlated measures and both independent predictors of vaccine hesitancy [26]. In addition, consistent with previous literature [45,46], our survey found that individuals who perceived to have a high risk of contracting COVID-19 were more inclined to accept the vaccination. As expected, risk perception played an important role in the uptake of the vaccination policy.

It is commonly believed that trust in the information and in those who deliver the information are key factors in achieving an effective communication strategy. As demonstrated by Larson H.J. et al., trust in government has a significant impact on vaccine uptake, underscoring the complex interplay between public trust and health behavior choices [47]. However, trust in government itself is a complex construct. In our research, we have specifically explored trust in the perceived transparency with which the government releases information to the public and found a positive association with vaccine acceptance. We found that people who perceived the government as transparent in its communication efforts were significantly more inclined to accept the vaccine and less susceptible to endorse misinformation about immunization. This aligns with previous research that has shown that a low level of trust in institutions and belief in misinformation are connected to a reduced likelihood of adopting recommended health behaviors [27,48,49,50,51,52]. Our analysis shows that when people view the government as transparent in their information and communication, they were more likely to be vaccine acceptant. The mechanisms used to disseminate government information can have a significant impact on its transparency. This includes the utilization of open government approaches, the availability of public documents, and the facilitation of open sessions and press conferences with the media [53].

Moreover, our study innovatively explores the endorsement of misinformation about the COVID-19 vaccine by developing a scale. While previous research has recognized misinformation as a significant factor in vaccine hesitancy [16,17,18,19,20], our work uniquely contributes to the limited literature on the determinants of misinformation endorsement, particularly among the US Spanish-speaking population. The scale we developed to measure this construct included questions on vaccine safety, efficacy, and transmission freedom of choice, as well as the perception of vaccination policies being used as political tools by the government. This scale proved to be predictive in determining individuals’ willingness to accept the COVID-19 vaccine based on their level of misinformation endorsement.

Our results showed that the determinants of misinformation endorsement are similar to the determinants of vaccine acceptance. We did not expect to find that those who used social media as their primary source of information were less likely to endorse unreliable information about the vaccine the more hours they spent on social networks. In this regard, the scientific literature has often associated the use of social media with the propagation of misinformation and increased hesitation towards the vaccine [20,54]. However, our study emphasizes the importance of understanding what people do on social media and which social media sources they have access to that could be protective and positively aligned with vaccine acceptance and eventually other public health preventive measures. For Facebook users only, we found a greater likelihood of misinformation endorsement. Furthermore, people who stated that information obtained through social media decreased their confidence in the COVID-19 vaccine, regardless of the amount of time spent on social media, confirm that social media use per se is not a negative behavior. It depends on other factors, such as what people do and what they search for, or what they are exposed to when they are using social media.

This social media finding leads us to the importance of understanding the role of digital health literacy in navigating and interpreting information on social media, as many studies have already shown how low literacy levels can impact vaccine confidence [55]. This underscores the necessity of focusing educational efforts on specific subgroups, regardless of their ethnicity, by implementing targeted communication and educational strategies aimed at enhancing people’s digital literacy based on their social media behaviors [53].

On the other hand, it is important to note that vaccination hesitancy cannot be attributed solely to the effectiveness of communication strategies. Our data show that among those who were willing to be vaccinated, one out of three did not have an appointment to do so. In our study, we did not investigate the reasons for these behaviors, but they could be associated with barriers to accessing vaccination sites, including long waiting times for appointments, concerns over immigration status repercussions, and transportation issues [56]. There exist initiatives aimed at bridging this gap and addressing these health needs. For example, public health leaders could address immigration concerns and transportation barriers by guaranteeing the confidentiality of immigration status, refraining from mandating proof of social security or legal residency for vaccination, organizing mobile vaccine events, and establishing long-term vaccination sites in neighborhoods with substantial Hispanic populations. Furthermore, providing information in Spanish about these locations could significantly contribute to these efforts [57].

## 5. Limitations

This study has several limitations. First, as a cross-sectional study, it cannot disentangle possible reverse causality between vaccine acceptance or misinformation endorsement and the studied independent variables. In addition, our sample did not include individuals who were mandated to be vaccinated and yet not fully convinced to do so as such we could not explore the factors associated with vaccine hesitancy in this group. In terms of outcomes, our study did not distinguish between different types of hesitancy and rejection of the vaccine. Regarding the responses we gathered, as in any survey focusing on highly polarizing issues such as vaccination behaviors, social desirability in the response is always possible. Another potential limitation regards the way we collected our data, the use of Pollfish biases the sample towards those familiar with using a mobile device. Pollfish offers financial compensation to mobile app developers for integrating the surveys into their applications. To motivate users to participate, minor monetary incentives are given to randomly selected individuals who finish the surveys. The Pollfish platform employs random device engagement (RDE) to connect with users actively using a mobile application, identifying them only by a unique device identifier [58].

Finally, when we measured the use of specific social media channels, respondents were not asked to select a particular channel exclusive of all others. Although this decision was made because it is unlikely that relatively social-media savvy individuals only use one channel, the consequence is that we were unable to assess the cumulative effect of getting information from multiple channels.

## 6. Conclusions

This paper demonstrates several avenues for public health interventions to boost vaccine uptake among Spanish speakers in the US, with an emphasis on the content and mechanisms of information dissemination, the potential positive role played by social media in the majority of the population, and the importance of maintaining government transparency when information is released to the public. Social media use was found to boost vaccine acceptance and diminish misinformation. Government transparency in vaccine communication is crucial for fostering and maintaining public trust. We developed a scale to measure COVID-19 vaccine misinformation endorsement and studied its association with vaccine acceptance. Vaccines play an ever-more important role in preventing severe illness and death among the most vulnerable. Improving understanding of the context- and population-specific drivers of vaccine hesitancy is crucial to promoting vaccine acceptance and increasing uptake as we pivot from pandemic to endemic disease response.

## Figures and Tables

**Table 1:** Sample characteristics by vaccine hesitant vs. acceptant respondents.

	Total (n = 483)		Hesitant (n = 297)		Acceptant (n = 186)		*p*-Value
Gender							0.62
Male	188	(38.9%)	113	(38.0%)	75	(40.3%)	
Female	295	(61.1%)	184	(62.0%)	111	(59.7%)	
Age category							0.84
18–24	94	(19.5%)	57	(19.2%)	37	(19.9%)	
25–34	97	(20.1%)	63	(21.2%)	34	(18.3%)	
35–44	97	(20.1%)	57	(19.2%)	40	(21.5%)	
45–54	126	(26.1%)	80	(26.9%)	46	(24.7%)	
54 and older	69	(14.3%)	40	(13.5%)	29	(15.6%)	
Geographic region							0.59
Midwest	47	(9.8%)	31	(10.5%)	16	(8.7%)	
Northeast	80	(16.7%)	53	(18.0%)	27	(14.8%)	
South	228	(47.7%)	140	(47.5%)	88	(48.1%)	
West	123	(25.7%)	71	(24.1%)	52	(28.4%)	
Educational attainment							0.99
High school or below	206	(43.4%)	127	(43.6%)	79	(42.9%)	
Some college	121	(25.5%)	74	(25.4%)	47	(25.5%)	
Bachelors or above	148	(31.2%)	90	(30.9%)	58	(31.5%)	
Food insecure past 3 months							0.046
Never	126	(26.1%)	89	(30.0%)	37	(19.9%)	
Sometimes	248	(51.3%)	146	(49.1%)	102	(54.8%)	
Often	109	(22.6%)	62	(20.9%)	47	(25.3%)	
Comorbidities	177	(36.7%)	110	(37.0%)	67	(36.0%)	0.82
Vaccinated for COVID-19							<0.001
Had 1 dose and plan to get 2nd	116	(24.0%)	51	(17.2%)	65	(34.9%)	
Had 1 dose, unsure if I’ll get 2nd	23	(4.8%)	9	(3.0%)	14	(7.5%)	
Had 1 dose, won’t get 2nd	2	(0.4%)	0	(0.0%)	2	(1.1%)	
Not yet, but I have an appt.	111	(23.0%)	62	(20.9%)	49	(26.3%)	
No, and I don’t have an appt.	231	(47.8%)	175	(58.9%)	56	(30.1%)	

**Table 2:** Simple and multivariable regression results as prevalence ratios (PRs) and 95% confidence intervals.

				Vaccine Acceptance vs. Hesitance				Misinformation Endorsement ****			
				Simple Model		Multiple Model **		Simple Model		Multiple Model **	
		n	%	PR	95% CI/ *p*-Value ***	PR	95% CI/ *p*-Value ***	PR	95% CI/ *p*-Value ***	PR	95% CI/ *p*-Value ***
COVID-19 risk perception score					*p* = 0.0001		*p* = 0.0005		*p* = 0.4284		
	Low risk	96	19.87%	ref	---	---	---	ref	---	---	---
	Middle risk	204	42.24%	1.37	0.94, 2.00	1.14	0.79, 1.65	0.79	0.55, 1.13		
	High risk	183	37.89%	2.01 *	1.39, 2.89	1.67 *^†^*	1.17, 2.40	0.85	0.57, 1.25		
Ever declined a recommended vaccine					*p* = 0.3519				*p* = 0.0254 ^#^		
	No	245	50.72%	ref	---			ref	---		
	Yes	152	31.47%	1.14	0.89, 1.45			1.50 *	1.09, 2.05		
	No recollection	86	17.81%	0.90	0.64, 1.26			1	0.64, 1.56		
Opinion about government transparency					*p* < 0.0001		*p* = 0.0022		*p* < 0.0001		*p* < 0.0001
	Not transparent	103	21.32%	ref	---	---	---	ref	---	---	---
	Moderately transparent	196	40.58%	2.15 *^†^*	1.42, 3.25	1.81 *^†^*	1.19, 2.76	0.50 *^†^*	0.36, 0.70	0.50 *^†^*	0.37, 0.69
	Very transparent	96	19.88%	2.66 *^‡^*	1.74, 4.06	2.21 *^†^*	1.44, 3.41	0.61 *	0.41, 0.90	0.73	0.50, 1.06
	I don’t know	88	18.22%	1.50	0.92, 2.47	1.49	0.91, 2.45	0.36 *^†^*	0.21, 0.60	0.42 *^†^*	0.25, 0.68
Time spent on social media					*p* = 0.8847		*p* = 0.4286 ^▲^		*p* < 0.6973		*p* = 0.4433 ^▲^
	Never	31	6.42%	ref	---	ref	---	ref	---	ref	---
	Not often	35	7.25%	0.89	0.47, 1.68	ref	---	0.89	0.38, 2.08	ref	---
	Every other day	63	13.04%	0.90	0.52, 1.57	ref	---	0.80	0.37, 1.73	ref	---
	Between 1–3 h per day	157	32.50%	1.07	0.66, 1.73	1.11	0.81, 1.52	1.16	0.61, 2.21	1.17	0.77, 1.78
	≥3 h per day	197	40.79%	0.98	0.61, 1.59	0.79	0.56, 1.10	1.06	0.56, 2.01	1.56	1.02, 2.37
No. of social media channels					*p* = 0.6266				*p* < 0.0034 ^#^		
	0 channels	129	26.71%	0.93	0.70, 1.29			1.24	0.83, 1.87		
	1 channel	131	27.12%	ref	---			Ref	---		
	2 channels	90	18.63%	1.18	0.85, 1.64			1.13	0.71, 1.79		
	3 channels	63	13.04%	1.21	0.85, 1.73			0.54	0.26, 1.10		
	4 channels	28	5.80%	1.17	0.72, 1.90			1.36	0.73, 2.52		
	5 channels	42	8.70%	0.97	0.61, 1.55			2.01 *^†^*	1.29, 3.13		
Trust vaccine information					*p* < 0.0001		*p* = 0.0008		*p* < 0.0001		*p* < 0.0001
	Low trust	228	47.20%	ref	---	---	---	ref	---	---	---
	Some trust	173	35.82%	1.53 *^†^*	1.16, 2.02	1.35 *	1.02, 1.79	0.39 *^‡^*	0.27, 0.57	0.45 *^‡^*	0.31, 0.65
	High trust	82	16.98%	2.33 *^‡^*	1.78, 3.05	1.76 *^†^*	1.31, 2.37	0.37 *^†^*	0.21, 0.63	0.50 *	0.29, 0.85
Social media impact on vaccine confidence					*p* = 0.0002 ^#^				*p* = 0.0017		*p* = 0.0148
	Increased	101	21.17%	ref	---			ref	---	---	---
	Reduced	73	15.30%	0.61 *^†^*	0.43, 0.87			2.11 *^†^*	1.33, 3.34	2.05 *^†^*	1.28, 3.30
	No change	160	33.54%	0.68 *^†^*	0.52, 0.88			1.29	0.82, 2.05	1.50	0.95, 2.37
	Unsure	49	10.27%	0.51 *^†^*	0.31, 0.81			0.69	0.31, 1.51	0.96	0.42, 2.20
	No information from social media	94	19.71%	0.51 *^†^*	0.35, 0.73			1.28	0.77, 2.13	1.33	0.77, 2.31
Misinformation endorsement score					*p* < 0.0001		*p* = 0.0031	NA		NA	
	First quartile (low endorsement)	110	22.77%	ref	---	ref	---				
	Second quartile	127	26.29%	0.46 *^‡^*	0.34, 0.63	0.59 *^†^*	0.44, 0.80				
	Third quartile	116	24.02%	0.55 *^‡^*	0.41, 0.73	0.77	0.57, 1.04				
	Fourth quartile (high endorsement)	130	26.92%	0.43 *^‡^*	0.31, 0.59	0.66 *	0.47, 0.92				
The following are binary predictors with the PR representing the prevalence of vaccine acceptance/misinformation endorsement in the people responding “yes” compared to those responding “no”.											
COVID-19 personal experience											
	Ever diagnosed with COVID-19	169	34.99%	1.07	0.85, 1.35			1.41 *	1.05, 1.88		
	Family or friend had COVID-19	256	53.00%	1.03	0.82, 1.29			0.97	0.72, 1.31		
	Family or friend died from COVID-19	98	20.29%	0.88	0.65, 1.18			0.94	0.64, 1.36		
Prior bad experience with vaccines		26	5.38%	0.49	0.22, 1.08			1.62 *	1.01, 2.61		
Distrust vaccines		59	12.21%	0.72	0.48, 1.10			2.06 *^‡^*	1.51, 2.82	1.88 *^†^*	1.33, 2.64
Main COVID-19 information source											
	Social media	147	30.43%	1.06	0.83, 1.35	0.57 ^▲^	0.25, 1.29	0.63 *	0.43, 0.91	1.39 ^▲^	0.58, 3.33
	Traditional media	425	87.99%	1.27	0.85, 1.90			1.05	0.66, 1.66		
	TV	351	72.67%	1.21	0.92, 1.59			1.11	0.79, 1.55		
	Radio	64	13.25%	0.97	0.69, 1.36			1.12	0.74, 1.69		
	Newspapers	106	21.95%	1.24	0.97, 1.59			0.93	0.65, 1.34		
	Traditional media (English)	408	84.47%	1.24	0.87, 1.76			1.01	0.67, 1.52		
	Traditional media (Spanish)	114	23.60%	1.16	0.90, 1.49			1.33	0.98, 1.83		
Vaccine info. from social media											
	No information from social media	94	19.46%	0.70 *	0.50, 0.99			0.99	0.68, 1.43		
	Facebook	241	49.90%	1.09	0.87, 1.37			1.37 *	1.02, 1.85	1.39 *	1.01, 1.92
	YouTube	182	37.68%	1.07	0.85, 1.34			1.21	0.90, 1.63		
	Twitter	101	20.91%	1.14	0.88, 1.48			1.13	0.80, 1.60		
	Instagram	170	35.20%	1.16	0.93, 1.46			1.01	0.74, 1.37		
	TikTok	128	26.50%	0.96	0.74, 1.25			0.94	0.67, 1.33		
Trust information from social media		113	23.40%	1.30 *	1.03, 1.66			0.98	0.69, 1.39		

* Significant at *p* = 0.05; *^†^* significant at *p* = 0.01; *^‡^* significant at *p* = 0.0001; ** adjusted for age, gender, and educational attainment; *** *p*-value from the contrast testing the overall significance of the predictor; ▲ interaction between amount of time spent on social media and social media as main COVID-19 information source significant at *p* = 0.05; ^#^ removed after being tested as non-significant predictor by the stepwise regression; **** the higher the score, the higher the misinformation endorsement.

## Data Availability

The data are available from the corresponding author upon reasonable request.

## Supplementary Materials

The following supporting information can be downloaded at https://www.mdpi.com/article/10.3390/healthcare12151545/s1. File S1: Complete survey in English and Spanish; File S2: Proportion of Respondents in Original and Recoded Response Categories for Misinformation Endorsement.
