# Supplementary material for: COVID-19 Vaccine Hesitancy and Misinformation Endorsement among a Sample of Native Spanish-Speakers in the US: A Cross-Sectional Study
Source: Healthcare (Basel). 2024 Aug 5;12(15):1545. doi: 10.3390/healthcare12151545 (PMC11311759; doi:10.3390/healthcare12151545)
Supplement: Supplementary file 1 [file healthcare-12-01545-s001.zip › healthcare-3076339-supplementary.pdf]

File S1

English version

1. **Have you taken the COVID-19 vaccine?**

○ Yes, I received two or more shots

○ Yes, I received one shot and the vaccine I took requires a second shot

○ Yes, I received one shot and the vaccine I took requires only one shot

○ Not yet, but I have an appointment scheduled for the first dose

○ No, and I do not have an appointment scheduled

2. **What vaccine/s are you eligible to receive? [check all that apply]**

- Pfizer BioNtech
- Moderna
- Johnson & Johson’s Jannsen
- Oxford-Astra-Zeneca
- Sputnik V
- Sinopharm (Beijing)
- Covishield (India)
- I do not know

3. **Are you a worker in any of the following categories? [select all that apply]**

❏ Healthcare worker

❏ Residential facility worker

❏ Public health worker

❏ Correctional facilities worker

❏ Vaccine manufacturing or distribution worker

❏ Pharmacy worker

❏ Teacher or school staff

❏ Food processing worker

❏ Grocery store worker

❏ Postal and shipping worker

❏ Transportation worker

❏ Police or firefighter

❏ Volunteer

❏ Other (please specify)

4. **What is your age category?**

- ○ 18-24
- ○ 25-34
- ○ 35-44
- ○ 45-54
- ○ 55-64
- ○ 65-74
- ○ 75+

5. **Sex:**

- ○ Male
- ○ Female

6. **Please select your state or territory of residence:**
   Use drop down menu with list of states and territories

7. **Are you a US citizen?**
   - Yes
   - No

8. **What race/ethnicity do you consider yourself?**

- ○ White, Non-Hispanic
- ○ Black, Non-Hispanic
- ○ Asian, Non-Hispanic
- ○ 2+ races
- ○ Hispanic
- ○ Prefer not to say
- ○ Other (please specify)

9. **What is the highest level of schooling you have completed?**

○ Less than high school

○ High school or equivalent

○ Some college

○ Bachelor's degree

○ Post-graduate degree (i.e. Master, PhD, MD, etc)

10. **In the past 12 months (1 year) are there been occasions in which you were worried about not having enough money or resources to be able to have enough food to eat?**

- Yes
- No

11. **Select the employment status that best describes your current situation [select one option only]:**

○ I am working - paid employee

○ I am working - self-employed

○ I am not working - on unemployment

○ I am not working - on paid leave or furloughed

○ I am not working - searching for work

○ I am retired

○ I am not working - on disability or worker's comp

○ I am not working - and not looking for a job

○ Other (please specify)

12. **Have you received or requested any of the following forms of support since the outbreak of COVID-19 pandemic?**

	Not requested or received	Have requested but have not yet received	Have requested but the request was rejected	Received
Unemployment benefit				
Wage support (supplement or replacement while still in employment or short-time working schemes)				
Paid sick leave or paid care leave (for example, for those who had to self-				

isolate or take care of children or dependent adults)				
State aid to businesses				
Other support from public services to help with living expenses or household needs (e.g. benefits, allowances, vouchers, food)				

13. **Do you have any of the following conditions? [check all that apply]**

- ❏ Cancer
- ❏ Severe allergies
- ❏ Seizures
- ❏ Immunocompromised state due to therapy or disease
- ❏ Autoimmune disease
- ❏ Overweight
- ❏ Obesity
- ❏ Diabetes (type 1 or 2)
- ❏ Cardiovascular disease
- ❏ Hypertension
- ❏ Pulmonary disease
- ❏ Rheumatological condition
- ❏ Pregnancy
- ❏ I do not have any medical condition
- ❏ Other (please specify)

14. * **How concerned are you about any of the following situations?:**

	Very concerned	Somewhat concerned	Not concerned
**Contracting COVID-19 at work?** (For example: your work settings that is not your home)	◯	◯	◯
**Contracting COVID-19 outside of work?** (For example: at the grocery store, when you are using transportation, or in other aspects of your daily life)	◯	◯	◯
**Infecting your family or friends with COVID-19?**	◯	◯	◯

Your experience ....

15. **Have you been diagnosed with COVID-19 by a healthcare worker or lab test result?**
    - ❏ Yes, over a year ago
    - ❏ Yes, in the past 12 months
    - ❏ No

16. **Did any of your close family members or friends experience any of the following? [Check all that apply]:**

- ❏ Tested positive for COVID-19 and had no symptoms or mild symptoms
- ❏ Tested positive for COVID-19 and had severe symptoms
- ❏ Died of COVID-19
- ❏ Lost their job or had a salary reduction due to COVID-19
- ❏ Got vaccinated for COVID-19 and My friends and family members who received the vaccine had a positive experience
- ❏ None of my close family or friends experience any of the above situations

17. **What do you think about the number of cases of COVID-19 reported in your country?**

- ○ The number of cases being reported is much lower than the actual number of cases
- ○ The number of cases being reported is much greater than the actual number of cases
- ○ The number of cases being reported is somewhat accurate
- ○ I don't know

18. **Do you feel you are receiving transparent information about the COVID-19 situation from your national government officials?**
    - ❏ Not at all
    - ❏ moderately transparent information
    - ❏ very transparent information
    - ❏ I do not know

19. **In your life, were you ever recommended a vaccine (other than the COVID-19 vaccine) by a healthcare provider that you did not take?**

- ○ Yes
- ○ No
- ○ I do not remember

20. **If you did not take the vaccine (other than COVID-19) that was recommended to you, what was/were the reason(s)? [check all reasons that applied to that situation]**

- ❏ I do not trust vaccines
- ❏ I did not think it was necessary
- ❏ I did not have enough information about the vaccine

- ❏ I did not think the vaccine was effective
- ❏ The vaccine was too expensive
- ❏ It was not logistically convenient to get the vaccine
- ❏ I did not think the vaccine was safe
- ❏ I was concerned about the side effects
- ❏ I had a prior bad experience with vaccinations
- ❏ I was afraid of needles
- ❏ For religious reasons
- ❏ Other reasons (please specify)

21. **Tell us how well the following statements describe your reactions and thoughts:**

	Very concerned	Somewhat concerned	Not concerned
I feel anxious when I see the number of COVID-19 cases climbing	◯	◯	◯
I find the prospect of a vaccine exciting	◯	◯	◯
I feel depressed about the uncertainty of how this pandemic will evolve	◯	◯	◯
I get upset when I hear contradictory information about COVID-19	◯	◯	◯
I feel stressed when I am unable to plan my life due to COVID-19	◯	◯	◯
I think that taking chances is part of life and so is taking the vaccine	◯	◯	◯

22. **How much time do you, on average, spend using social media?**

- Everyday for 3 hours or more
- Every day for more than 1 hour but less than 3 hours
- Alternate days
- Not often
- Never

Information about the vaccine...

23. **Did you get information about the COVID-19 vaccine from social media ? [check all that apply]**

- ❑ No, I did not
- ❑ I am not sure
- ❑ Yes - from Facebook
- ❑ Yes - from YouTube
- ❑ Yes - from Instagram
- ❑ Yes - from TikTok
- ❑ Yes - from Twitter
- ❑ Other social media (please specify which one)

Social media use ...

24. [skip logic following q23] Did the information you got from social media change your level of confidence in the COVID-19 vaccine?

- ○ Increased my confidence in the vaccine
- ○ Decreased my confidence in the vaccine
- ○ Did not change my confidence
- ○ I am not sure
- ○ Did not change my confidence but influenced my opinion in other ways - please specify

25. Did you ever share information on social media about the COVID-19 vaccine?

- ○ Yes
- ○ No

26. **When you read or hear something about the COVID-19 vaccine that concerns what source do you rely on for getting additional information? [check all that apply]**

- ❑ Family members
- ❑ My doctor
- ❑ My friends
- ❑ Social media
- ❑ TV
- ❑ I do my own research online
- ❑ I do nothing
- ❑ other ---------------------------------

Trust in information...

27. **From what source did you get the most information about the COVID-19 vaccine? Select up to 3 SOURCES:**

1. Local television news (on TV or on the web)
2. English language national or cable network news (on TV or on the web)
3. Non-English language television station (on TV or on the web)
4. National newspaper (i.e. New York Times, Wall Street Journal, USA Today on paper or on the web)
5. My town or other local newspaper (on paper or on the web)
6. Non-English language newspaper (on paper or on the web)
7. English language radio
8. Non-English language radio
9. News portal site such as Yahoo! or MSN
10. Website of a government agency
11. Social media
12. Word of mouth
13. Through my employer
14. Other (please specify)

28. **How much do you <u>trust the information</u> you got so far about the COVID-19 vaccine?**

○ Not at all

○ Very little

○ Somewhat

○ A lot

29. **Who would you trust the most to give you information about the COVID-19 vaccine in the near future? Select your <u>TOP 3 choices</u>:**

❏ National officials

❏ Your local government leaders

❏ Public health experts

❏ Your employer

❏ Your co-workers

❏ Your doctor

❏ Your local pharmacy

❏ Your family and friends

❏ Your community health center

❏ A celebrity (for example: a sports figure, actor, or musician)

❏ Local leaders in your community not in government positions (for example: local organizations, religious leaders)

❏ Other (please specify)

Your opinions ...

30. **I think that most of the measures taken so far by the US government to respond to the COVID-19 pandemic have been:**
    - ❑ Just right
    - ❑ Excessive
    - ❑ Not useful
    - ❑ Counter-productive
    - ❑ I don't know

31. **If you were offered a COVID-19 vaccine - at no cost to you- how likely are you to take it?**

- ○ Very likely
- ○ Somewhat likely
- ○ I am not sure
- ○ Somewhat unlikely
- ○ Very unlikely
- ○ I would not take it at the moment but would consider it later on

Confidence...

32. How much do you agree or disagree with the following statements?:

1 – Strongly disagree

2 – Disagree

3 – Somewhat disagree

4 – Unsure

5 – Somewhat agree

6 – Agree

7 – Strongly agree

- ❑ You cannot get COVID-19 from the vaccine itself
- ❑ There are no toxic ingredients in the vaccine that can harm your health
- ❑ The vaccine cannot mess up your DNA
- ❑ The vaccine cannot cause infertility
- ❑ The vaccine cannot cause other diseases
- ❑ The fast production of the vaccine did not compromise its safety
- ❑ The vaccine is not going to be used by Governments as a tool to limit our civil rights (right of assembly, right of movement, right of religion, etc.)

33. **How much do you agree or disagree with the following statements?:**

1 – Strongly disagree

2 – Disagree

3 – Somewhat disagree

4 – Unsure

5 – Somewhat agree

6 – Agree

7 – Strongly agree

- ❏ The vaccine will work in protecting me from getting COVID-19
- ❏ By taking the vaccine I will protect my friends and family from getting COVID-19
- ❏ Everyone should get the vaccine to achieve herd immunity
- ❏ Getting COVID-19 is worse than experiencing potential side effects from the vaccine
- ❏ Natural remedies will not protect me from COVID-19

34. **How much do you agree or disagree with the following statements?:**

1 – Strongly disagree

2 – Disagree

3 – Somewhat disagree

4 – Unsure

5 – Somewhat agree

6 – Agree

7 – Strongly agree

- ❏ People should be free to decide if getting vaccinated or not with no consequences for their job or personal life
- ❏ People should have the option to choose the vaccine brand they want
- ❏ People should be allowed to live their life with no restrictions once vaccinated
- ❏ Healthcare professionals and scientists with concerns about the vaccine should have opportunities to share their opinions with the public
- ❏ Everybody should have equal access to the most effective and safe vaccine regardless of income, race, or immigration status
- ❏ There is no elite group that will achieve financial power if people are getting vaccinated
- ❏ There is no microchip with tracking capabilities inserted in the vaccine

35. If you have other opinions about the vaccine you would like to share, please write them here;

Spanish version

1. **Se ha vacunado contra el COVID-19?**

○ Sí, recibí dos o más dosis

○ Sí recibí una dosis y la vacuna que recibí requiere una segunda dosis

○ Sí, recibí una dosis y la vacuna que recibí requiere solo una dosis

○ Aún no, pero tengo una cita programada para la primera dosis

○ No, y no tengo una cita programada

2. **¿Para cuál vacuna es elegible? [Marque todas las que correspondan]**

- Pfizer BioNtech
- Moderna
- Johnson & Johson's Jannsen
- Oxford-Astra-Zeneca
- Sputnik V
- Sinopharm (Beijing)
- Covishield (India)
- No lo sé

3. **¿Es un trabajador en alguna de las siguientes categorías? [Seleccione todas las opciones que correspondan]**

❏ Trabajador de la atención médica

❏ Trabajador de instalación residencial

❏ Trabajador de salud pública

❏ Trabajador de instalaciones correccionales

❏ Trabajador de la fabricación o distribución de vacunas

❏ Trabajador de farmacia

❏ Docente o personal escolar

❏ Trabajador de procesamiento de alimentos

❏ Trabajador de tienda de comestibles

❏ Trabajador del servicio postal y envíos

❏ Trabajador del servicio de transporte

❏ Policía o bombero

❏ Voluntario

❏ Otro (especifique)

4. ¿En qué categoría se encuentra su edad?

- ○ 18-24
- ○ 25-34
- ○ 35-44
- ○ 45-54
- ○ 55-64
- ○ 65-74
- ○ +75

5. Sexo:

- ○ Masculino
- ○ Femenino

6. Seleccione su estado o territorio de residencia:

7. ¿Es ciudadano estadounidense?

- Sí
- No

8. ¿Qué raza/etnia considera que tiene usted?

- ○ Blanca, no hispana
- ○ Negra, no hispana
- ○ Asiática, no hispana
- ○ +2 razas
- ○ Hispana
- ○ Prefiere no decir
- ○ Otra (especifique)

9. ¿Cuál es el nivel más alto de escolaridad que ha completado?

- ○ Inferior al de secundaria
- ○ Secundaria o equivalente
- ○ Un poco de universidad
- ○ Licenciatura
- ○ Postgrado (ej. Maestría, PhD, MD, etc.)

10. En los últimos 12 meses (1 año), ¿ha habido ocasiones en las que le ha preocupado no tener suficiente dinero o recursos para poder tener suficiente comida para comer?

- Sí
- No

11. Seleccione el estado de empleo que <u>mejor describe</u> su situación actual [seleccione una opción solamente]:

- ○ Estoy trabajando: empleado con pago
- ○ Estoy trabajando: independiente
- ○ No estoy trabajando: desempleado
- ○ No estoy trabajando: con licencia pagada o ausente con permiso
- ○ No estoy trabajando: buscando empleo
- ○ Estoy en jubilación
- ○ No estoy trabajando: por discapacidad o compensación laboral
- ○ No estoy trabajando y no estoy buscando trabajo
- ○ Otro (especifique)

12. ¿Ha recibido o solicitado alguna de las siguientes formas de apoyo desde el brote de la pandemia de COVID-19?

	Sin solicitar o recibir	Lo he solicitado, pero aún no lo he recibido	Hice la solicitud, pero fue rechazada	Lo he recibido
Beneficio de desempleo				
Apoyo salarial (complemento o reemplazo mientras aún está en el empleo o en planes de trabajo de jornada reducida)				
Licencia pagada por enfermedad o licencia con atención pagada (por ejemplo, para quienes tuvieron que aislarse o cuidar a niños o adultos dependientes)				

Ayudas estatales a las empresas				
Otro apoyo de los servicios públicos para ayudar con los gastos de subsistencia o las necesidades del hogar (por ejemplo, prestaciones, asignaciones, vales, alimentos)				

13. ¿Tiene alguna de las siguientes condiciones? [Marque todas las que correspondan]

- ❏ Cáncer
- ❏ Alergias graves
- ❏ Convulsiones
- ❏ Estado inmunodeprimido debido a terapia o enfermedad
- ❏ Enfermedad autoinmune
- ❏ Sobrepeso
- ❏ Obesidad
- ❏ Diabetes (tipo 1 o 2)
- ❏ Enfermedad cardiovascular
- ❏ Hipertensión
- ❏ Enfermedad pulmonar
- ❏ Condición reumatológica
- ❏ Embarazo
- ❏ No tengo ninguna condición médica
- ❏ Otro (especifique)

14. *¿Qué tan preocupado está por alguna de las siguientes situaciones?:

	Muy preocupado	Algo preocupado	No estoy preocupado
**¿Contraer COVID-19 en el trabajo?** (por ejemplo: su entorno de trabajo que no es su hogar)	◯	◯	◯
**¿Contraer COVID-19 fuera del trabajo?** (por ejemplo: en el supermercado, cuando usa el transporte o en otros aspectos de su vida diaria)	◯	◯	◯
**¿Infectar a su familia o amigos con COVID-19?**	◯	◯	◯

15. ¿Un trabajador de salud o el resultado de una prueba de laboratorio le ha sido diagnosticado con COVID-19?

- ❏ Sí, hace más de un año
- ❏ Sí, en los últimos 12 meses
- ❏ No

16. ¿Alguno de sus familiares o amigos cercanos experimentó alguno de los siguientes síntomas? [Marque todos los que correspondan]:

- ❏ Dio positivo para COVID-19 y no presentó síntomas o tuvo síntomas leves
- ❏ Dio positivo para COVID-19 y tuvo síntomas graves
- ❏ Murió de COVID-19
- ❏ Perdió su trabajo o su salario fue reducido debido al COVID-19
- ❏ Me vacuné contra el COVID-19 y mis amigos y familiares que recibieron la vacuna tuvieron una experiencia positiva
- ❏ Ninguno de mis familiares o amigos cercanos ha experimentado alguna de las situaciones anteriores

17. ¿Qué piensa de la cantidad de casos de COVID-19 notificados en su país?

- ○ El número de casos que se notifican es mucho menor que el número real de casos
- ○ El número de casos que se notifican es mucho mayor que el número real de casos
- ○ La cantidad de casos notificados es algo precisa
- ○ No sé

18. ¿Siente que está recibiendo información transparente sobre la situación del COVID-19 de parte de los funcionarios de su gobierno nacional?

- ❏ Para nada
- ❏ Información moderadamente transparente
- ❏ Información muy transparente
- ❏ No lo sé

19. En su vida, ¿alguna vez un proveedor de atención médica le recomendó una vacuna (además de la vacuna del COVID-19) que no tomó?

- ○ Sí
- ○ No
- ○ No recuerdo

20. Si no se puso la vacuna (que no sea del COVID-19) que se le recomendó, ¿cuál fue/fueron las razones? [Marque todas las razones que corresponden a esa situación]

- ❏ No confío en las vacunas
- ❏ No creía que fuera necesaria
- ❏ No tenía suficiente información sobre la vacuna
- ❏ No pensé que la vacuna fuera efectiva

❑ La vacuna era demasiado cara
❑ No era logísticamente conveniente recibir la vacuna
❑ No pensé que la vacuna fuera segura
❑ Me preocupaban los efectos secundarios
❑ Tuve una mala experiencia previa con las vacunas
❑ Tuve miedo a las agujas
❑ Por motivos religiosos
❑ Otras razones (especifique)

21. Díganos qué tan bien describen sus reacciones y pensamientos las siguientes declaraciones:

	Muy preocupado	Algo preocupado	No preocupado
Me siento ansioso cuando veo que aumenta el número de casos de COVID-19	◯	◯	◯
Encuentro emocionante la perspectiva de una vacuna	◯	◯	◯
Me deprime la incertidumbre sobre cómo evolucionará esta pandemia	◯	◯	◯
Me enojo cuando escucho información contradictoria sobre el COVID-19	◯	◯	◯
Me estreso cuando no puedo planificar mi vida debido al COVID-19	◯	◯	◯
Creo que arriesgarse forma parte de la vida y también lo es recibir la vacuna	◯	◯	◯

22. ¿En promedio, cuánto tiempo pasa usando las redes sociales?

- Todos los días durante 3 horas o más
- Todos los días durante más de 1 hora, pero menos de 3 horas.
- Días alternos
- No a menudo
- Nunca

23. ¿Obtuvo información sobre la vacuna del COVID-19 en las redes sociales? [Marque todas las opciones que correspondan]

- ❏ No, no la obtuve
- ❏ No estoy seguro
- ❏ Sí, de Facebook
- ❏ Sí, de YouTube
- ❏ Sí, de Instagram
- ❏ Sí, de TikTok
- ❏ Sí, de Twitter
- ❏ Otras redes sociales (especifique cuál)

24. ¿La información que obtuvo de las redes sociales cambió su nivel de confianza en la vacuna del COVID-19?

- ○ Aumenté mi confianza en la vacuna
- ○ Disminuyó mi confianza en la vacuna
- ○ No cambió mi confianza
- ○ No estoy seguro
- ○ No cambió mi confianza, pero influyó en mi opinión de otras formas; especifique

25. ¿Compartió alguna vez información en las redes sociales sobre la vacuna del COVID-19?

- ○ Sí
- ○ No

26. Cuando lee o escucha algo sobre la vacuna COVID-19 que es preocupante, ¿en qué fuente confía para obtener información adicional? [Marque todas las que correspondan]

- ❏ Miembros de la familia
- ❏ Mi doctor
- ❏ Mis amigos
- ❏ Redes sociales
- ❏ TV
- ❏ Hago mi propia búsqueda en línea
- ❏ No hago nada
- ❏ Otra ---------------------------------

27. ¿De qué fuente obtuvo la mayor información sobre la vacuna del COVID-19? Seleccione hasta 3 FUENTES:

14. Noticias en la televisión local (en TV o en la web)
15. Noticias nacionales o de cadenas de cable en inglés (en televisión o en la web)
16. Canal de televisión en un idioma diferente al inglés (en la televisión o en la web)
17. Periódico nacional (es decir, New York Times, Wall Street Journal, USA Today en papel o en la web)
18. Periódico de mi ciudad u otro periódico local (en papel o en la web)
19. Periódico en otro idioma (en papel o en la web)
20. Radio en inglés
21. Radio en idiomas distintos del inglés
22. Un sitio de portal de noticias como Yahoo! o MSN
23. Sitio web de una agencia gubernamental
24. Redes sociales
25. Boca a boca
26. A través de mi empleador

14. Otro (especifique)

28. ¿Cuánto confía en la información que ha adquirido hasta ahora sobre la vacuna del COVID-19?

○ Nada en lo absoluto

○ Muy poco

○ Un poco

○ Bastante

29. ¿En quién confiaría más para que le brinde información sobre la vacuna del COVID-19 en un futuro cercano? Seleccione sus <u>3 opciones PRINCIPALES</u>:

❏ Funcionarios nacionales
❏ Los líderes de su gobierno local
❏ Expertos en salud pública
❏ Su empleador
❏ Sus compañeros de trabajo
❏ Su médico
❏ Su farmacia local
❏ Su familia y amigos
❏ Su centro de salud comunitario
❏ Una celebridad (por ejemplo: un deportista, actor o músico)
❏ Líderes locales de su comunidad que no ocupan puestos gubernamentales (por ej. organizaciones locales, líderes religiosos)
❏ Otro (especifique)

30. **Creo que la mayoría de las medidas tomadas hasta ahora por el gobierno de Estados Unidos para responder a la pandemia del COVID-19 ha sido:**
    - ❏ Apropiadas
    - ❏ Excesivas
    - ❏ No son útiles
    - ❏ Contraproducente
    - ❏ No lo sé

31. **Si le ofrecieran una vacuna del COVID-19 sin ningún costo para usted, ¿qué probabilidades hay de que la reciba?**

- ○ Muy probable
- ○ Algo probable
- ○ No estoy seguro
- ○ Algo improbable
- ○ Muy improbable
- ○ No la recibiría en este momento, pero la consideraría más adelante

32. **¿En qué medida está de acuerdo o en desacuerdo con las siguientes afirmaciones?:**

1 – Muy en desacuerdo

2 - En desacuerdo

3 - Algo en desacuerdo

4 - No estoy seguro

5 - Algo de acuerdo

6 - De acuerdo

7 - Totalmente de acuerdo

- ❏ No puede contraer COVID-19 a través de la vacuna
- ❏ No hay ingredientes tóxicos en la vacuna que puedan dañar su salud
- ❏ La vacuna no puede alterar su ADN
- ❏ La vacuna no puede causar infertilidad
- ❏ La vacuna no puede causar otras enfermedades
- ❏ La rápida producción de la vacuna no comprometió su seguridad
- ❏ Los gobiernos no van a utilizar la vacuna como una herramienta para limitar nuestros derechos civiles (derecho de reunión, derecho de circulación, derecho de religión, etc.)

33. **¿En qué medida está de acuerdo o en desacuerdo con las siguientes afirmaciones?:**

1 – Muy en desacuerdo

2 – En desacuerdo

3 – Algo en desacuerdo

4 – No estoy seguro

5 – Algo de acuerdo

6 – De acuerdo

7 – Muy de acuerdo

- ❏ La vacuna funcionará para protegerme de contraer COVID-19
- ❏ Al recibir la vacuna, protegeré a mis amigos y familiares de contraer COVID-19.
- ❏ Todos deben recibir la vacuna para lograr la inmunidad colectiva.
- ❏ Contraer COVID-19 es peor que experimentar los posibles efectos secundarios de la vacuna
- ❏ Los remedios naturales no me protegerán del COVID-19

34. ¿En qué medida está de acuerdo o en desacuerdo con las siguientes afirmaciones?:

1 – Muy en desacuerdo

2 - En desacuerdo

3 - Algo en desacuerdo

4 - No estoy seguro

5 - Algo de acuerdo

6 - De acuerdo

7 - Totalmente de acuerdo

- ❏ Las personas deben tener la libertad de decidir si se vacunan o no sin consecuencias para su trabajo o su vida personal
- ❏ Las personas deben tener la opción de elegir la marca de vacuna que desean recibir
- ❏ Se debe permitir que las personas vivan su vida sin restricciones una vez vacunadas
- ❏ Los profesionales de la salud y los científicos que tengan inquietudes sobre la vacuna deben tener la oportunidad de compartir sus opiniones con el público
- ❏ Todo el mundo debería tener el mismo acceso a la vacuna más eficaz y segura, independientemente de sus ingresos, raza o estado migratorio
- ❏ No existe ningún grupo de élite que alcance el poder financiero cuando las personas se vacunen
- ❏ La vacuna no contiene un microchip con capacidad de seguimiento

35. Si tiene otras opiniones sobre la vacuna que le gustaría compartir, escríbalas aquí;

File S2

Table S1 shows the proportion of respondents in each of the seven original response categories and the recoded three-category responses for the seven questions used to measure misinformation endorsement.

Table S1. Misinformation Endorsement Questions

	Original Response Categories (%)							Recoded Responses* (%)		
	**Strongly disagree**	**Disagree**	**Somewhat disagree**	**Unsure**	**Somewhat agree**	**Agree**	**Strongly agree**	**Disagree**	**Unsure**	**Agree**
1. You cannot get COVID-19 from the vaccine itself	18.0	14.1	9.9	31.3	8.1	11.6	7.0	42.0	31.3	26.7
2. There are no toxic ingredients in the vaccine that can harm your health	17.8	9.9	11.0	34.4	9.7	9.9	7.3	38.7	34.4	26.9
3. The vaccine cannot mess up your DNA	12.6	9.3	11.2	37.5	7.9	12.4	9.1	33.1	37.5	29.4
4. The vaccine cannot cause infertility	14.9	9.5	7.7	37.3	10.6	12.4	7.7	32.1	37.3	30.6
5. The vaccine cannot cause other diseases	15.3	13.9	12.0	33.3	9.1	10.6	5.8	41.2	33.3	25.5
6. The fast production of the vaccine did not compromise its safety	14.3	11.6	11.6	25.9	16.6	11.8	8.3	37.5	25.9	36.6
7. The vaccine is not going to be used by Governments as a tool to limit our civil rights (right of assembly, right of movement, right of religion, etc.)	16.4	11.6	11.6	31.1	11.8	8.7	8.9	39.5	31.1	29.4

* Original response categories recoded as “Strongly Disagree”, “Disagree” and “Somewhat disagree” equals “Disagree”, “Unsure” was unchanged and “Strongly agree”, “Agree” and “Somewhat agree” equals “Agree”.
